# Tight Spaces, Big Discoveries: Decoding Human Adhesion Biology with Avian Chorioallantoic Membrane Xenograft Models

**DOI:** 10.3390/cancers18030508

**Published:** 2026-02-03

**Authors:** Niamh McAuley, Izabela Cymer, Robyn Stanley, Sinead Toomey, Catriona M. Dowling, Albert Leung, Ann M. Hopkins, Cathy E. Richards

**Affiliations:** 1 Department of Surgery, RCSI University of Medicine and Health Sciences, D09 YD60 Dublin, Ireland; izabeladrozdz@rcsi.ie (I.C.); annhopkins@rcsi.ie (A.M.H.); 2 Department of Medicine, RCSI University of Medicine and Health Sciences, D09 YD60 Dublin, Ireland; robynstanley22@rcsi.ie (R.S.); sineadtoomey@rcsi.ie (S.T.); catrionadowling@rcsi.ie (C.M.D.); 3 School of Dentistry, RCSI University of Medicine and Health Sciences, D18 NY72 Dublin, Ireland; albertleung@rcsi.ie

**Keywords:** tight junctions, JAM-A, chorioallantoic membrane, cancer progression, tumour microenvironment, angiogenesis, patient-derived xenografts, 3Rs

## Abstract

Tight junction (TJ) proteins, such as Junctional Adhesion Molecule-A (JAM-A), claudins, and occludin, play increasingly recognized roles in cancer biology beyond their structural functions, influencing tumour proliferation, invasion, metastasis and therapy resistance. Understanding how these proteins modulate tumour progression in vivo requires models that are both physiologically relevant and ethically viable. The chick chorioallantoic membrane (CAM) xenograft model has emerged as a powerful and cost-effective in vivo system that aligns with the 3Rs (replacement, reduction, and refinement), offering unique advantages such as vascular accessibility, rapid tumour growth kinetics and immunotolerance. This review explores how the CAM model can be leveraged to study the mechanistic role of TJ proteins in tumour–stroma interactions, angiogenesis, extracellular matrix (ECM) remodelling and mechanotransduction, including the YAP/TAZ pathway. While limitations remain, particularly with respect to immune modelling and long-term studies, recent advances in imaging, genetic manipulation and integration of patient-derived xenografts (PDXs) are expanding the model’s translational relevance. Standardizing methodologies and embracing new molecular tools will further elevate the utility of this approach as a complementary platform to traditional rodent models, with significant promise for TJ-focused cancer research and therapeutic innovation.

## Simple Summary

Tight junction proteins, including JAM-A, claudins, and occludin, are known for maintaining tissue barriers, but they also influence how cancers grow, spread, and respond to treatment. Studying these proteins in living systems requires models that are realistic, fast, and ethically responsible. The chick chorioallantoic membrane (CAM) model offers a cost-effective way to observe tumour growth, blood vessel formation, and interactions with surrounding tissue in a short timeframe. This review highlights how the CAM model can help researchers understand the role of tight junction proteins in cancer progression, including their effects on tissue structure, cell signalling, and tumour invasiveness. By combining this model with new imaging techniques, genetic tools, and patient-derived samples, scientists can gain faster and more detailed insights into cancer biology. Using the CAM alongside traditional rodent models could improve preclinical research and support the development of new therapies targeting tight junction proteins.

## 1. Introduction

### 1.1. Introduction to the CAM Model

Xenografting technology has revolutionized the translation of basic cell biology studies into higher organisms, with the evolution of patient-derived xenografting (PDX) complementing the large-scale omic studies that have driven precision medicine approaches in cancer [1]. Although rodents are most commonly used as tumour-bearing xenograft models, there are other models that offer advantages in cost, scalability and compliance with the 3R principles (replacement, reduction, and refinement). The chick embryo chorioallantoic membrane (CAM) xenograft model was first used as a host for tumour cells in 1911 [2]. Since then, it has become a widely used alternative model for the study of tumour formation [3,4], angiogenesis [5,6], invasion [7], metastasis [8], proliferation [9] and therapeutic intervention [10].

The CAM is an extra-embryonic structure that forms in avian eggs through the fusion of the chorion and the allantois membranes between embryonic development day (EDD) 3.75-EDD4 [11]. The membrane is composed of three distinct layers, the ectoderm, mesoderm, and endoderm, each contributing to essential functions such as respiratory gas exchange, calcium resorption and metabolic waste removal, serving as a functional analogue to the mammalian placenta [11,12]. Its most remarkable feature is the rapid development of an extensive capillary network, rendering it a highly vascularized tissue that supports the avian embryo housed within the egg [13,14]. This vascularity, combined with the relatively transient immunological naiveté of the embryo [15], has positioned the CAM model as a powerful tool for studying tumour biology across a range of cancer subtypes.

Over the past several decades, the CAM protocol has gained significant traction in cancer research due in part to this early immunological naiveté, which permits the growth of human tumour xenografts with a relatively low risk of tumour rejection [16]. A variety of biological materials, including conditioned medium [17], immortalized cell lines [3], primary blood samples [18] or tissues [14], and organoids [19], have been successfully introduced into this model. When implanted onto the CAM surface, tumour cells readily form three-dimensional masses that recapitulate key features of solid tumour behaviour [20]. The short embryonic developmental window (7–10 days) following tumour cell implantation enables rapid data collection, while the external accessibility of the CAM facilitates real-time imaging, repeated drug administration and histological sampling—all without the need for surgical intervention. These advantages make the CAM model particularly valuable as an intermediate system that bridges the gap between in vitro experiments and more complex mammalian in vivo models. Additionally, CAM experiments are performed at a fraction of the cost of traditional murine models, often 98% cheaper, depending on the type of mouse used [21].

### 1.2. Studying the Hallmarks of Cancer in the CAM Model

One of the primary strengths of the CAM model lies in its ability to support multifaceted investigations into the hallmarks of cancer [9]. Angiogenesis, the formation of new blood vessels from pre-existing vasculature, is readily induced by tumour xenografts (or even tumour-derived soluble material) on the CAM and can be quantified via gross visualization or histological staining. Vessel density, branching patterns and convergence toward the tumour mass are frequently used as endpoints [22]. Accordingly, the model greatly supports the evaluation of anti-angiogenic agents, which can be administered topically or intravenously via the CAM vasculature, allowing for real-time assessment of drug efficacy [14].

Tumour invasion into the CAM stroma is another key application of the model, with the ectodermal and mesodermal layers providing structural integrity that permits the evaluation of cellular motility and tissue infiltration. Immunohistochemistry using human-specific cytokeratin antibodies enables visualization of invasive fronts, while serial sectioning permits quantification of invasion depth and area. Additionally, matrix metalloproteinase activity and degradation of the basement membrane can be assessed to elucidate mechanisms of invasion [23].

The CAM model is also well suited for studying metastatic dissemination, a process that cannot be recapitulated in vitro. Tumour cells that enter the vasculature can be detected at secondary sites, including distal regions of the CAM and embryonic organs such as the liver, lungs, and brain. These metastatic events can be confirmed using molecular techniques such as PCR amplification of human-specific Alu sequences or by imaging fluorescently labelled cells in situ [6,8,24].

Proliferation, a fundamental aspect of tumour progression and therapeutic responsiveness, can be assessed in the CAM model using markers such as Ki-67, which identifies actively cycling cells [25]. In addition, Western blotting and immunofluorescence can be employed to assess the activation of proliferation-related signalling pathways, such as MAPK/ERK, offering a molecular readout that complements morphological findings [26].

Inflammation is rapidly triggered post-grafting, with heterophils (avian neutrophil equivalents) and monocytes/macrophages infiltrating within hours [24]. These cells release cytokines and matrix metalloproteinases (MMPs), such as MMP-9 from heterophils and MMP-13 from macrophages, driving extracellular matrix remodelling. This inflammatory milieu promotes angiogenesis and compromises endothelial integrity. Tight junction proteins—including claudins and ZO family members—are particularly susceptible to MMP activity and cytokine signalling. The CAM model thus offers a dynamic system to study how inflammation-driven MMPs contribute to cancer-associated vascular remodelling via tight junction disruption and barrier dysfunction.

The chick CAM model presents a versatile, ethically advantageous, and cost-effective platform for the comprehensive study of tumours, both common and rare. Its ability to model angiogenesis, invasion, metastasis and proliferation in a dynamic, vascularized environment—combined with its utility in novel drug evaluation and barrier biology—makes it an indispensable tool in modern cancer research. This is particularly true when interrogating the emerging functional role of tight junction (TJ) adhesion complexes in tumour progression.

### 1.3. Adhesion, Tight Junctions (TJs) and the CAM Model

TJs are the apical-most component of epithelial and endothelial intercellular junctional complexes, establishing a selective, semi-permeable barrier that regulates the paracellular movement of ions, solutes and macromolecules. Increasingly, it is recognized that the physiological functions of TJs extend beyond their role in maintaining barrier integrity [27,28]. TJs are critical regulators of cell polarity; contribute to the maintenance of tissue architecture; and are involved in intracellular signalling pathways that influence cell proliferation [29], differentiation, and migration and play a role in stem cell maintenance [30].

**Figure 1:**
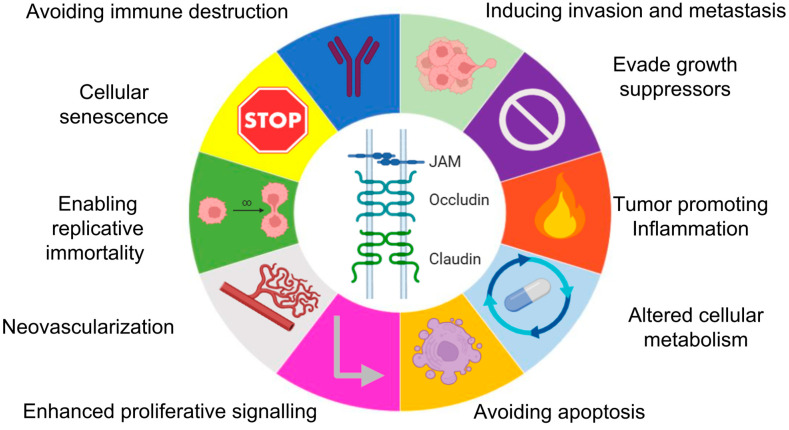
Participation of TJs in the hallmarks of cancer. TJ components, including JAMs, occludin, and claudins, are traditionally known for regulating epithelial and endothelial barrier function. However, emerging evidence highlights their broader roles in oncogenesis. Dysregulation of TJ proteins has been implicated in multiple cancer hallmarks, including sustained proliferative signalling, evasion of growth suppressors, resistance to cell death and deregulated metabolism. In addition, TJs influence inflammation, immune evasion, cellular senescence, neovascularization and metastatic dissemination. These diverse functions position TJ proteins as key regulators of tumour initiation, progression and resistance to therapy. Publication license: https://BioRender.com/5yzcs3y accessed on 29 January 2026.

Importantly, the dysregulation of TJ components, including both transmembrane proteins such as claudins, occludin and Junctional Adhesion Molecules (JAMs), as well as cytoplasmic scaffolding partners like ZO-1, has been implicated in various aspects of cancer biology [31]. Altered expression, mislocalization, or functional disruption of TJ proteins can promote loss of cell polarity, epithelial-to-mesenchymal transition (EMT), increased invasiveness, neovascularization, evasion of cell death and metastatic dissemination [32]—recognizable events within the Hallmarks of Cancer (Figure 1).

In the context of the CAM xenograft model—a well-established in vivo platform for studying tumour progression and regression, angiogenesis [33,34], invasion and metastasis [10,35,36]—the potential contribution of TJs to these events is of particular relevance. The highly-vascularized and physiologically relevant environment of the CAM model enables observation of dynamic TJ remodelling in response to tumour cell grafting or pro-invasive stimuli. Understanding how TJ proteins contribute to, or are altered during, tumour–host interactions in the CAM model offers valuable insights into the roles of TJs in tumour progression and as targets of synthetic inhibitors or actors in the function of immunotherapy drugs.

This review will explore the multifaceted roles of TJ proteins in tumour pathophysiology, highlighting their contributions to oncogenic processes, as elucidated through CAM-based studies, and discussing their potential utility as biomarkers or therapeutic targets in solid tumours.

## 2. Results

### 2.1. Integral Membrane TJ Proteins

Junctional Adhesion Molecules (JAMs): Junctional Adhesion Molecule (JAM) proteins are members of the immunoglobulin superfamily of adhesion receptors. Among this superfamily, JAM-A is the most extensively studied in the context of cancer biology. Early work suggested that loss of JAM-A expression was associated with increased migratory potential in breast cancer cells [37]. However, subsequent studies have increasingly indicated that JAM-A overexpression, rather than its loss, is more commonly associated with aggressive tumour phenotypes [38,39,40,41]. This reflects the complex nature of TJ proteins in either suppressing or promoting tumourigenesis, depending on the cellular context, and has opened up its investigation as a potential therapeutic target.

**Figure 2:**
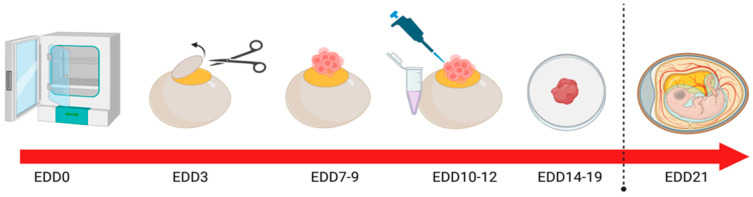
The CAM xenograft model as a platform for investigating the role of tight junctions in cancer progression. In vivo methodology: At EDD0, eggs are cleaned and placed in an incubator with high humidity. At EDD3, a small window is created in the eggshell to assess embryo viability; viable embryos are resealed with semi-permeable tape. Between EDD7 and 9, tumour cells are implanted in the CAM surface, allowing xenografts to develop undisturbed over the subsequent 72 h. At EDD10-12, treatments are applied. The protocol is terminated between EDD14 and 19; the tumour is excised and sent for downstream analysis. Chicks naturally hatch at EDD21, and all experimentation must cease before this timepoint. Publication license: https://BioRender.com/gucx0nw accessed on 29 January 2026.

CAM modelling has been successfully used to interrogate the role of JAM-A in HER2-positive breast cancer in vivo using the methodology outlined in (Figure 2). Specifically, a recombinant fragment of the extracellular domain of JAM-A (rsJAM-A) was found to significantly increase the invasion of human SK-BR-3 cells implanted in the CAM, as quantified by pan-cytokeratin staining deep into the intermediate mesodermal layer [42]. Additionally, Ki67 positivity was also increased in rsJAM-A-treated xenografts compared to controls. Moreover, the size of rs-JAM-treated xenografts was grossly larger than vehicle control-treated tumours [42].

In related work, the natural antibiotic Tetrocarcin A was found to reduce JAM-A expression in human HER2-positive breast cells in vitro, while sublethal doses reduced gross tumour size and proliferation index in HER2-positive model breast tumours grown on the CAM [43]. The same compound also induced a pro-apoptotic response via the upregulation of cleaved caspase-3 expression in triple-negative breast tumours in the CAM model [44]. While targeted protein reductions can also be achieved via gene silencing in tumour cells prior to/after implantation upon the CAM, it bears mentioning that transient silencing may be insufficient to influence global tumour phenotypes. Nonetheless, one study on JAM-A silencing in CAM-grown model breast tumours exhibited significantly altered staining of the proliferation marker Ki67 despite a very low siRNA transfection efficiency. Similarly, in a CAM model of gastro-oesophageal cancer using the methodology outlined in (Figure 2), transient JAM-A silencing resulted in heterogeneous expression patterns that closely mirrored the intra-tumoural variability observed in patient-derived tumours [45].

Moreover, a *cis*-dimerization inhibitor of JAM-A, termed JBS2, has been shown to reduce the number of macroscopically visible HER2-positive model breast tumours on the CAM without causing overt embryonic toxicity, echoing parallel results from preclinical mouse models [36]. Pairing JBS2 with the HER tyrosine kinase inhibitor lapatinib evoked a more pronounced reduction in macroscopic tumour size in ovo, but at the price of increased embryonic death [36].

The role of JAM-A in angiogenesis is well established [46,47,48], and its contribution to neovascularization in multiple myeloma has been recently studied in the CAM model. Specifically, Solimando and colleagues found an increase in angiogenesis when rJAM-A was added to MM endothelial cells in ovo [49], and, correspondingly, angiogenesis was impaired by an inhibitory JAM-A monoclonal antibody (J10.4). These findings were further validated in MM-bearing mouse models, suggesting that impairing *homo*-dimerization of JAM-A restricts angiogenesis and represents a druggable target in MM [50]. Notably, the increased overall survival of patients with MM has allowed for the emergence and detection of aggressive extramedullary disease (EMD), which may have previously gone unrecognized due to earlier mortality. EMD is often characterized by a distinct gene expression profile enriched for epithelial-to-mesenchymal transition (EMT) and focal adhesion pathways, both features being closely tied to TJ dynamics and metastatic potential [51]. Given the short developmental timeframe of the chick embryo (21-day gestation), the CAM xenograft model offers an excellent platform for investigating metastatic lesions or EMD in a timely manner.

To further underscore the significance of the JAM family in carcinogenesis, JAM-A has recently emerged as a potential immune-modulatory factor in human tumours [52]. The chick embryo is uniquely equipped with a developing but functional immune system [15,53] and offers a novel platform to investigate immune cell infiltration and tumour responses to immunotherapy. This presents a distinct advantage over traditional rodent models [15,54,55], which often require immunosuppression to permit tumour engraftment, thereby limiting their utility in assessing immune-related therapeutic responses. Building upon compelling in silico and in vitro evidence across multiple tumour types [52], future studies may explore the potential of molecularly targeting JAM-A and co-treating with an immunotherapy for in vivo validation within an immunocompetent model using similar methods to (Figure 2). This approach holds promise for identifying novel therapeutic strategies and enhancing understanding of tumour-immune interactions in a physiologically relevant setting. The immune system of pre- and post-treatment developing embryos can be evaluated in a number of ways, as outlined in Table 1.

Occludin and Claudins: Besides JAM-A, the role of other TJ proteins in cancer have also been studied in the CAM model. Occludin and claudins are integral membrane proteins that form the structural backbone of TJ architecture and are essential for maintaining barrier integrity. Beyond their architectural roles, they are also involved in regulating signalling in pathways that influence tumour progression [56].

For example, Growth Differentiation Factor 11 (GDF11), a key regulator of cellular differentiation, has been shown to impair the invasive capacity of hepatocarcinoma cells in vitro by modulating the expression of genes associated with TJ and EMT, including *occludin* [57]. Building on these findings, researchers employed the CAM model to investigate the effects of recombinant human GDF11 on tumour invasion. Liver xenograft tumours treated with GDF11 exhibited limited invasive behaviour, with the majority of implanted tumour cells confined to the surface of the CAM [57]. However, untreated xenografts displayed a notable presence of highly proliferative cells in the lower CAM layers, consistent with deeper tumour invasion in the absence of GDF11 treatment [57].

Claudins, too, have been studied in the context of angiogenesis in the CAM model. LPS-induced inflammation increases angiogenesis and upregulates tight junction genes, including claudin-1, claudin-5, and claudin-12. Treatment with the antioxidant NAC reverses these effects, normalizing claudin expression and reducing vascular permeability, while also increasing VE-cadherin expression to potentially restore endothelial junction stability [58]. Tight junction permeability measurement is outlined in Table 1.

### 2.2. Other Adhesion Complexes and Signalling TJ Proteins

YAP and TAZ: The TJ-affiliated signalling proteins Yes-associated protein (YAP) and transcriptional co-activator with PDZ-binding motif (TAZ), effectors of the Hippo signalling pathway, have emerged as critical regulators of tumour angiogenesis, invasion, and metastasis. Their activity is closely modulated by mechanical cues and extracellular matrix stiffness—features that can be precisely manipulated and visualized in the CAM model [12,59]. In this context, YAP/TAZ have been shown to influence tumour angiogenesis by regulating pro-angiogenic factors such as CTGF, CYR61, angiopoietin-2 and VEGF [60,61] while also modulating endothelial cell function and vascular remodelling [62]. The clinical significance of YAP/TAZ dysregulation in human cancers is underscored by accumulating evidence linking aberrant TJ protein expression to poor prognosis, enhanced metastasis, and therapeutic resistance across multiple tumour types. Although the broader role of YAP/TAZ in cancer cell invasion and metastasis is well established, few studies have directly examined these mechanisms using the CAM model. Notably, Jiang et al. (2020) demonstrated enhanced invasion of triple-negative breast cancer cells via YAP signalling in a CAM xenograft model, highlighting its utility for mechanistic investigation [63]. The suitability of this model for functional perturbation makes it valuable for testing YAP/TAZ-targeted strategies and understanding their mechanosensitive role in the tumour microenvironment. As such, the CAM model presents a physiologically relevant system for advancing our understanding of YAP/TAZ-driven cancer progression.

Cadherins: Cadherins, particularly E- and N-cadherin, are calcium-dependent adhesion molecules forming part of the adherens junctional complex. They play a crucial role in maintaining cell–cell adhesion and linking the cell membrane to the actin cytoskeleton. Their dysregulation is closely associated with EMT and metastatic dissemination.

In a non-small cell lung cancer (NSCLC) model using the CAM, the nicotinic acetylcholine receptor subunit alpha 5 (α5-nAChR) and lymphocyte antigen 6 complex, locus E (Ly6E), were shown to influence tumour behaviour via the TGF-β1/Smad signalling pathway [64]. While initial in vitro experiments demonstrated their role in promoting cancer cell migration, their functional relevance was confirmed in vivo on the CAM, where siRNA-mediated knockdown of α5-nAChR or Ly6E led to reduced expression of EMT markers such as ZEB1 and vimentin, as assessed by immunohistochemistry [64]. Although E-cadherin expression was not directly measured in the CAM model, the downregulation of ZEB1—a known repressor of E-cadherin—raises the possibility that restoring E-cadherin could re-establish epithelial adhesion and counteract EMT in these tumours. Additional studies have also identified promising drug combinations (such as Phenethyl Isothiocyanate and Dasatinib) that limited E-cadherin and N-cadherin expression in hepatocellular carcinoma in vitro and blunted angiogenesis in vivo in a CAM model [65]. Future investigations into the expressional changes in cadherin proteins in vivo could provide further mechanistic insight into their role in tumour progression and angiogenesis methods, as outlined in Table 1.

Integrins: The integrins are a group of transmembrane receptors that mediate cell adhesion to the ECM and play pivotal roles in cell signalling and the hallmarks of cancer. Recent work has explored the therapeutic modulation of integrins in glioblastoma multiforme in the CAM model. For instance, co-treatment with natural compounds berbamine and arcyriaflavin A (ArcA) at concentrations that significantly reduced the levels of integrin α6 and key stem cell markers (Sox2, Nanog, Oct4) in vitro was also shown to reduce tumour weight in vivo, highlighting the potential of integrin targeting in glioblastoma [66]. Other integrin subunits, such as β_1_ and β_3_, have also been shown to cooperate with VEGFR receptors to induce angiogenesis. Integrins have been underexplored in the CAM model to date, but its vascularized and immunocompetent environment offers a promising platform to investigate tools such as a novel PEG-cRGD-conjugated drug called BGC0222, which has already been shown in a CAM setting to have antitumour activity via binding to *avβ3* and blunting neovascularization [67].

**Table 1:** Downstream methods for evaluating hallmarks of oncogenesis in the CAM.

Hallmark Being Evaluated	Protocol	Reference
Evaluating cell state and morphology	Immunohistochemistry	[44]
	Western blotting	[25]
	Haematoxylin and eosin	[10,26]
Assessing neovascularization	Gross imaging of vasculature	[22,49]
	Evans blue dye	[58]
	Yolk sac membrane	[65]
Proliferation and inflammation	Weighing tumour mass	[25]
	Ki67 staining	[68]
	ELISA	[6,69,70]
	Fluorescent-tagged cells	[24]
	Cytokeratin staining	[23,42,57]
	MRI for secondary tumour sites	[71,72]
	Alu sequence PCRs	[8]
Altered metabolism	Analysis of proteome	[73,74]
Immune response of the embryo	Flow cytometry	[9]

## 3. Discussion

The chick chorioallantoic membrane (CAM) model continues to emerge as a powerful, physiologically relevant in vivo system for investigating tumour biology. This in vivo model is a highly convenient, accessible, reliable, and cost-effective alternative, aligning well with the 3R principles of minimizing animal experimentation—replacement, reduction and refinement. Importantly, in many jurisdictions, experimentation using the chick chorioallantoic membrane (CAM) model does not require formal ethical approval, as the embryo is not considered sentient or innervated prior to hatching. However, regulatory requirements may vary internationally and should be confirmed on a case-by-case basis. Its unique advantages, including low cost, rapid tumour growth kinetics and immunotolerance early in development, make it an attractive and ethically favourable complement to mammalian models and well suited to studying the complex interplay between tumour cells and their microenvironment in an accelerated experimental timeline. Their alignment with the 3R principles also supports more sustainable and humane research practices. In the UK, a review of Home Office animal licences from 2017 to 2023 identified eight licence applications for angiogenesis research that collectively proposed to use 135,000 mice over 5 years [75]. Given the highly vascularized nature of the CAM and its established utility as a model for angiogenesis research and drug testing [9], the case to use it as an intermediate model that reduces animal use is compelling.

Crucially, the CAM model’s accessibility, vascular richness and compatibility with live imaging afford a dynamic platform for studying not just angiogenesis but also tumour cell invasion and extracellular matrix (ECM) interactions in real time. This is further enhanced by its ability to support the growth of a wide range of tumour types, from established cell lines to, increasingly, patient-derived xenografts (PDXs). The ease with which experimental manipulations and live imaging can be undertaken underscores its flexibility and translational potential, particularly when compared to traditional rodent models.

In the context of tight junction (TJ) proteins, such as JAM-A, occludin, and claudins, the CAM model is particularly well positioned to dissect their multifaceted roles in tumour progression. These proteins do not merely function as structural barriers; they actively regulate signalling pathways implicated in proliferation, metastasis, and therapy resistance. The short gestation period of the chick embryo and its responsiveness to genetic or pharmacological manipulation allow for the rapid evaluation of TJ-mediated phenotypes in vivo. Moreover, the emerging connection between junctional integrity and mechanotransduction pathways, particularly through YAP/TAZ signalling, adds further value to the CAM model. As mechanical cues from the tumour microenvironment (TME) are increasingly recognized as central regulators of cancer behaviour, the CAM provides a biomechanically responsive environment to interrogate these interactions at both the molecular and tissue levels. Although studies investigating signalling proteins using the CAM model remain limited, this system should be considered a promising in vivo platform for future tumour biology research in this area.

Despite these strengths, the model has inherent limitations, outlined in Table 2. The embryonic nature of the chick host means that aspects of the immune–tumour interaction cannot be fully recapitulated, and the absence of mature stromal components may influence tumour growth kinetics or drug response. Additionally, the short experimental window, typically 7–10 days post-engraftment, limits the assessment of long-term outcomes such as dormancy, late-stage metastasis, the development of clinical drug resistance or tumour recurrence post-resection. Reproducibility can also be challenged by inter-operator variability in grafting techniques that might alter overall tumour take rates or embryo viability.

Nonetheless, recent methodological innovations are addressing many of these limitations. Advances in live imaging, quantitative image analysis and molecular profiling (e.g., spatial transcriptomics or multiplex immunofluorescence) now enable high-resolution mapping of tumour–stroma interactions within the CAM either in vivo or, particularly, in ex ovo models [76,77]. The integration of CRISPR/Cas9 and RNAi technologies into CAM workflows further facilitates functional genomic screening, enabling the dissection of gene-specific roles in tumour growth and invasion. Moreover, the growing use of PDXs within the CAM is accelerating the development of rapid ex vivo platforms for precision oncology, allowing tumour-specific responses to be evaluated in a matter of days, bridging the gap between bench and bedside.

To further enhance the impact of CAM-based research, greater standardization of protocols, including tumour implantation techniques, vessel quantification methods and timing of interventions, is essential. Establishing consensus guidelines and reporting standards would improve reproducibility and promote wider adoption across oncology research disciplines.

## 4. Conclusions

As oncology research increasingly shifts toward preclinical models that are physiologically relevant but also ethically responsible and cost-effective, the CAM model is poised to play an increasingly prominent role. Its utility extends beyond traditional angiogenesis assays to encompass mechanistic exploration of tumour progression, with particular promise in studying cell junction dynamics, ECM interactions and mechanosensitive pathways such as YAP/TAZ. Standardization of experimental protocols, coupled with advances in imaging and molecular analyses, will be key to enhancing the reproducibility and translational relevance of this model.

Importantly, the CAM does not aim to entirely replace murine xenograft models but to complement them. Its unique strengths—particularly in early-stage screening, angiogenesis studies and potential for mechanistic dissection of individual protein contributions in cancer—provide critical insights that can guide and refine more complex in vivo studies. In the context of TJ signalling and its intersection with related pathways, the CAM offers a tractable and insightful platform for unravelling how structural proteins orchestrate tumour progression. When used alongside traditional models, the CAM model enriches the preclinical research toolkit, helping to faster translate molecular insights into therapeutic innovation.

In conclusion, the CAM model represents a critical asset in the preclinical cancer research landscape. Its strategic use can enhance mechanistic discovery, reduce reliance on mammalian models, and contribute to the acceleration of therapeutic development, particularly in fields focused on TME dynamics and cell junctional signalling.

## Figures and Tables

**Table 2:** Strengths and limitations of the chick chorioallantoic membrane (CAM) xenograft model in cancer research.

Strengths	Limitations
Rapid tumour growth: Allows for evaluation of tumour progression, angiogenesis, and invasion within 7–10 days.	Short experimental window: Limited to ~7–10 days post-engraftment, restricting long-term studies.
High vascularity: Enables real-time assessment of neovascularization and vascular targeting therapies.	Immature immune system: Lacks full adaptive immunity, limiting study of immunotherapy or tumour–immune interactions.
Cost-effective and low-maintenance: Inexpensive compared to murine models; requires minimal infrastructure.	Species differences: Avian host may not fully recapitulate mammalian tumour–stroma or ECM interactions.
No need for immunosuppression: Natural immune deficiency in the embryo supports xenograft engraftment.	Variable tumour take rates: Can suffer from inter-operator variability and inconsistent tumour establishment.
Ethically favourable: Not classified as an animal experiment in many jurisdictions (<ED14), aligning with the 3Rs.	Limited stromal complexity: Absence of fully developed mammalian stromal compartments may affect tumour behaviour.
Supports diverse tumour types: Compatible with cell lines; organoids; and, increasingly, patient-derived xenografts (PDXs).	Lack of long-term metastatic model: While early invasion and dissemination can be studied, full metastatic colonization is limited.
Accessible for imaging and manipulation: Translucent membrane allows for direct observation and microinjection.	Limited availability of chick-specific reagents: Antibodies and molecular tools for host–tumour interaction studies are less developed.
Amenable to genetic and pharmacological modulation: Supports siRNA, CRISPR, and small-molecule screening.	Reproducibility challenges: Standardization of protocols and quantification methods is still evolving.
Translational potential: Emerging role in personalized oncology through PDX and drug response studies.	Not suitable for chronic or immune-mediated diseases: Inadequate for studying long-term tumour–host dynamics.

## Data Availability

Not applicable. No datasets were generated or analysed for this review.

## Abbreviations

The following abbreviations are used in this manuscript:

CAM	Chorioallantoic Membrane
TJ	Tight Junction
JAM-A	Junctional Adhesion Molecule-A
PDX	Patient-Derived Xenograft
ECM	Extracellular Matrix
TME	Tumour Microenvironment
YAP	Yes-associated Protein
TAZ	Transcriptional Co-Activator with PDZ-binding Motif
RNAi	RNA Interference
CRISPR	Clustered Regularly Interspaced Short Palindromic Repeats

## References

[1] Kim S.-Y., van de Wetering M., Clevers H., Sanders K. (2025). The future of tumor organoids in precision therapy. Trends Cancer.

[2] Rous P. (1911). A SARCOMA OF THE FOWL TRANSMISSIBLE BY AN AGENT SEPARABLE FROM THE TUMOR CELLS. J. Exp. Med..

[3] Kunz P., Schenker A., Sahr H., Lehner B., Fellenberg J. (2019). Optimization of the chicken chorioallantoic membrane assay as reliable in vivo model for the analysis of osteosarcoma. PLoS ONE.

[4] Jaworski S., Sawosz E., Grodzik M., Kutwin M., Wierzbicki M., Włodyga K., Jasik A., Reichert M., Chwalibog A. (2013). Comparison of tumour morphology and structure from U87 and U118 glioma cells cultured on chicken embryo chorioallantoic membrane. Bull. Vet. Inst. Pulawy.

[5] Ribatti D., Nico B., Vacca A., Roncali L., Burri P.H., Djonov V. (2001). Chorioallantoic membrane capillary bed: A useful target for studying angiogenesis and anti-angiogenesis in vivo. Anat. Rec..

[6] Steiner N., Ribatti D., Willenbacher W., Jöhrer K., Kern J., Marinaccio C., Aracil M., García-Fernández L.F., Gastl G., Untergasser G. (2015). Marine compounds inhibit growth of multiple myeloma in vitro and in vivo. Oncotarget.

[7] Ranjan R.A., Muenzner J.K., Kunze P., Geppert C.I., Ruebner M., Huebner H., Fasching P.A., Beckmann M.W., Bauerle T., Hartmann A. (2023). The Chorioallantoic Membrane Xenograft Assay as a Reliable Model for Investigating the Biology of Breast Cancer. Cancers.

[8] Zijlstra A., Mellor R., Panzarella G., Aimes R.T., Hooper J.D., Marchenko N.D., Quigley J.P. (2002). A Quantitative Analysis of Rate-limiting Steps in the Metastatic Cascade Using Human-specific Real-Time Polymerase Chain Reaction. Cancer Res..

[9] Fischer D., Fluegen G., Garcia P., Ghaffari-Tabrizi-Wizsy N., Gribaldo L., Huang R.Y., Rasche V., Ribatti D., Rousset X., Pinto M.T. (2022). The CAM Model-Q&A with Experts. Cancers.

[10] Aslam R., Richards C.E., Fay J., Hudson L., Workman J., Lee C.L., Murphy A., O’Neill B., Toomey S., Hennessy B.T. (2024). Synergistic Effects of the Combination of Alpelisib (PI3K Inhibitor) and Ribociclib (CDK4/6 Inhibitor) in Preclinical Colorectal Cancer Models. Int. J. Mol. Sci..

[11] Nagai H., Tanoue Y., Nakamura T., Chan C.J.J., Yamada S., Saitou M., Fukuda T., Sheng G. (2022). Mesothelial fusion mediates chorioallantoic membrane formation. Philos. Trans. R. Soc. Lond. B Biol. Sci..

[12] Nowak-Sliwinska P., Segura T., Iruela-Arispe M.L. (2014). The chicken chorioallantoic membrane model in biology, medicine and bioengineering. Angiogenesis.

[13] Ribatti D. (2017). The chick embryo chorioallantoic membrane (CAM) assay. Reprod. Toxicol..

[14] Chen L., Wang S., Feng Y., Zhang J., Du Y., Zhang J., Ongeval C.V., Ni Y., Li Y. (2021). Utilisation of Chick Embryo Chorioallantoic Membrane as a Model Platform for Imaging-Navigated Biomedical Research. Cells.

[15] Garcia P., Wang Y., Viallet J., Macek Jilkova Z. (2021). The Chicken Embryo Model: A Novel and Relevant Model for Immune-Based Studies. Front. Immunol..

[16] Ribatti D. (2016). The chick embryo chorioallantoic membrane (CAM). A multifaceted experimental model. Mech. Dev..

[17] Vacca A., Ribatti D., Presta M., Minischetti M., Iurlaro M., Ria R., Albini A., Bussolino F., Dammacco F. (1999). Bone Marrow Neovascularization, Plasma Cell Angiogenic Potential, and Matrix Metalloproteinase-2 Secretion Parallel Progression of Human Multiple Myeloma. Blood.

[18] Pizon M., Schott D., Pachmann U., Schobert R., Pizon M., Wozniak M., Bobinski R., Pachmann K. (2022). Chick Chorioallantoic Membrane (CAM) Assays as a Model of Patient-Derived Xenografts from Circulating Cancer Stem Cells (cCSCs) in Breast Cancer Patients. Cancers.

[19] Bencurova K., Tran L., Friske J., Bevc K., Helbich T.H., Hacker M., Bergmann M., Zeitlinger M., Haug A., Mitterhauser M. (2024). An in vivo tumour organoid model based on the chick embryonic chorioallantoic membrane mimics key characteristics of the patient tissue: A proof-of-concept study. EJNMMI Res..

[20] Wang J., Wang L., Cai L. (2009). Establishment of a transplantation tumor model of human osteosarcoma in chick embryo. Chin.-Ger. J. Clin. Oncol..

[21] Hu J., Ishihara M., Chin A.I., Wu L. (2019). Establishment of xenografts of urological cancers on chicken chorioallantoic membrane (CAM) to study metastasis. Precis. Clin. Med..

[22] Ribatti D. (2012). Chicken Chorioallantoic Membrane Angiogenesis Model. Methods Mol. Biol..

[23] Deryugina E.I., Zijlstra A., Partridge J.J., Kupriyanova T.A., Madsen M.A., Papagiannakopoulos T., Quigley J.P. (2005). Unexpected effect of matrix metalloproteinase down-regulation on vascular intravasation and metastasis of human fibrosarcoma cells selected in vivo for high rates of dissemination. Cancer Res..

[24] Deryugina E.I., Quigley J.P. (2008). Chick embryo chorioallantoic membrane model systems to study and visualize human tumor cell metastasis. Histochem. Cell Biol..

[25] Skowron M.A., Sathe A., Romano A., Hoffmann M.J., Schulz W.A., van Koeveringe G.A., Albers P., Nawroth R., Niegisch G. (2017). Applying the chicken embryo chorioallantoic membrane assay to study treatment approaches in urothelial carcinoma. Urol. Oncol..

[26] Shekatkar M., Kheur S., Deshpande S., Sakhare S., Sanap A., Kheur M., Bhonde R. (2024). Critical appraisal of the chorioallantoic membrane model for studying angiogenesis in preclinical research. Mol. Biol. Rep..

[27] Kakogiannos N., Ferrari L., Giampietro C., Scalise A.A., Maderna C., Rava M., Taddei A., Lampugnani M.G., Pisati F., Malinverno M. (2020). JAM-A Acts via C/EBP-alpha to Promote Claudin-5 Expression and Enhance Endothelial Barrier Function. Circ. Res..

[28] Zammarchi I., Santacroce G., Puga-Tejada M., Hayes B., Crotty R., O’Driscoll E., Majumder S., Kaczmarczyk W., Maeda Y., McCarthy J. (2024). Epithelial neutrophil localization and tight junction Claudin-2 expression are innovative outcome predictors in inflammatory bowel disease. United Eur. Gastroenterol. J..

[29] Leech A.O., Cruz R.G., Hill A.D., Hopkins A.M. (2015). Paradigms lost-an emerging role for over-expression of tight junction adhesion proteins in cancer pathogenesis. Ann. Transl. Med..

[30] Dionísio M.R., Vieira A.F., Carvalho R., Conde I., Oliveira M., Gomes M., Pinto M.T., Pereira P., Pimentel J., Souza C. (2020). BR-BCSC Signature: The Cancer Stem Cell Profile Enriched in Brain Metastases that Predicts a Worse Prognosis in Lymph Node-Positive Breast Cancer. Cells.

[31] Anderson J.M., Van Itallie C.M. (2009). Physiology and function of the tight junction. Cold Spring Harb. Perspect. Biol..

[32] Hanahan D. (2022). Hallmarks of Cancer: New Dimensions. Cancer Discov..

[33] Wan Z., Hirche C., Fricke F., Dragu A., Will P.A. (2025). Chick Chorioallantoic Membrane as an in vivo Model for the Study of Angiogenesis and Lymphangiogenesis. J. Vasc. Res..

[34] Ribatti D. (2022). The chick embryo chorioallantoic membrane as an experimental model to study in vivo angiogenesis in glioblastoma multiforme. Brain Res. Bull..

[35] Lokman N.A., Elder A.S.F., Ricciardelli C., Oehler M.K. (2012). Chick chorioallantoic membrane (CAM) assay as an in vivo model to study the effect of newly identified molecules on ovarian cancer invasion and metastasis. Int. J. Mol. Sci..

[36] Smith Y.E., Wang G., Flynn C.L., Madden S.F., MacEneaney O., Cruz R.G.B., Richards C.E., Jahns H., Brennan M., Cremona M. (2022). Functional Antagonism of Junctional Adhesion Molecule-A (JAM-A), Overexpressed in Breast Ductal Carcinoma In Situ (DCIS), Reduces HER2-Positive Tumor Progression. Cancers.

[37] Naik M.U., Naik T.U., Suckow A.T., Duncan M.K., Naik U.P. (2008). Attenuation of junctional adhesion molecule-A is a contributing factor for breast cancer cell invasion. Cancer Res..

[38] Brennan K., McSherry E.A., Hudson L., Kay E.W., Hill A.D.K., Young L.S., Hopkins A.M. (2013). Junctional adhesion molecule-A is co-expressed with HER2 in breast tumors and acts as a novel regulator of HER2 protein degradation and signaling. Oncogene.

[39] Murakami T., Takasawa A., Takasawa K., Akimoto T., Aoyama T., Magara K., Saito Y., Ota M., Kyuno D., Yamamoto S. (2021). Aberrant expression of junctional adhesion molecule-A contributes to the malignancy of cervical adenocarcinoma by interaction with poliovirus receptor/CD155. Cancer Sci..

[40] McSherry E.A., Brennan K., Hudson L., Hill A.D.K., Hopkins A.M. (2011). Breast cancer cell migration is regulated through junctional adhesion molecule-A-mediated activation of Rap1 GTPase. Breast Cancer Res..

[41] Lathia J.D., Li M., Sinyuk M., Alvarado A.G., Flavahan W.A., Stoltz K., Rosager A.M., Hale J., Hitomi M., Gallagher J. (2014). High-Throughput Flow Cytometry Screening Reveals a Role for Junctional Adhesion Molecule A as a Cancer Stem Cell Maintenance Factor. Cell Rep..

[42] Leech A.O., Vellanki S.H., Rutherford E.J., Keogh A., Jahns H., Hudson L., O’Donovan N., Sabri S., Abdulkarim B., Sheehan K.M. (2018). Cleavage of the extracellular domain of junctional adhesion molecule-A is associated with resistance to anti-HER2 therapies in breast cancer settings. Breast Cancer Res..

[43] Vellanki S.H., Cruz R.G.B., Jahns H., Hudson L., Sette G., Eramo A., Hopkins A.M. (2019). Natural compound Tetrocarcin-A downregulates Junctional Adhesion Molecule-A in conjunction with HER2 and inhibitor of apoptosis proteins and inhibits tumor cell growth. Cancer Lett..

[44] Vellanki S.H., Cruz R.G.B., Richards C.E., Smith Y.E., Hudson L., Jahns H., Hopkins A.M. (2019). Antibiotic Tetrocarcin-A Down-regulates JAM-A, IAPs and Induces Apoptosis in Triple-negative Breast Cancer Models. Anticancer Res..

[45] Richards C.E., Sheehan K.M., Kay E.W., Hedner C., Borg D., Fay J., O’Grady A., Hill A.D.K., Jirstrom K., Hopkins A.M. (2021). Development of a Novel Weighted Ranking Method for Immunohistochemical Quantification of a Heterogeneously Expressed Protein in Gastro-Esophageal Cancers. Cancers.

[46] Kummer D., Ebnet K. (2018). Junctional Adhesion Molecules (JAMs): The JAM-Integrin Connection. Cells.

[47] Ebnet K. (2017). Junctional Adhesion Molecules (JAMs): Cell Adhesion Receptors With Pleiotropic Functions in Cell Physiology and Development. Physiol. Rev..

[48] Shu F., Lu J., Zhang W., Huang H., Lin J., Jiang L., Liu W., Liu T., Xiao S., Zheng Y. (2023). JAM-A Overexpression in Human Umbilical Cord-Derived Mesenchymal Stem Cells Accelerated the Angiogenesis of Diabetic Wound By Enhancing Both Paracrine Function and Survival of Mesenchymal Stem Cells. Stem Cell Rev. Rep..

[49] Solimando A.G., Da Via M.C., Leone P., Borrelli P., Croci G.A., Tabares P., Brandl A., Di Lernia G., Bianchi F.P., Tafuri S. (2021). Halting the vicious cycle within the multiple myeloma ecosystem: Blocking JAM-A on bone marrow endothelial cells restores angiogenic homeostasis and suppresses tumor progression. Haematologica.

[50] Solimando A.G., Brandl A., Mattenheimer K., Graf C., Ritz M., Ruckdeschel A., Stühmer T., Mokhtari Z., Rudelius M., Dotterweich J. (2018). JAM-A as a prognostic factor and new therapeutic target in multiple myeloma. Leukemia.

[51] Solimando A.G., Da Via’ M.C., Borrelli P., Leone P., Di Lernia G., Tabares Gaviria P., Brandl A., Pedone G.L., Rauert-Wunderlich H., Lapa C. (2018). Central Function for JAM-a in Multiple Myeloma Patients with Extramedullary Disease. Blood.

[52] Ren T., Zheng Y., Liu F., Liu C., Zhang B., Ren H., Gao X., Wei Y., Sun Q., Huang H. (2024). Identification and Validation of JAM-A as a Novel Prognostic and Immune Factor in Human Tumors. Biomedicines.

[53] Miebach L., Berner J., Bekeschus S. (2022). In ovo model in cancer research and tumor immunology. Front. Immunol..

[54] Nipper A.J., Warren E.A.K., Liao K.S., Liu H.-C., Michikawa C., Porter C.E., Wells G.A., Villanueva M., Brasil da Costa F.H., Veeramachaneni R. (2024). Chick Embryo Chorioallantoic Membrane as a Platform for Assessing the In Vivo Efficacy of Chimeric Antigen Receptor T-cell Therapy in Solid Tumors. ImmunoHorizons.

[55] Hartle S., Sutton K., Vervelde L., Dalgaard T.S. (2024). Delineation of chicken immune markers in the era of omics and multicolor flow cytometry. Front. Vet. Sci..

[56] Vellanki S.H., Richards C., Smith Y., Hopkins A. (2016). The Contribution of Ig-Superfamily and MARVEL D Tight Junction Proteins to Cancer Pathobiology. Curr. Pathobiol. Rep..

[57] Gerardo-Ramirez M., Lazzarini-Lechuga R., Hernandez-Rizo S., Jimenez-Salazar J.E., Simoni-Nieves A., Garcia-Ruiz C., Fernandez-Checa J.C., Marquardt J.U., Coulouarn C., Gutierrez-Ruiz M.C. (2019). GDF11 exhibits tumor suppressive properties in hepatocellular carcinoma cells by restricting clonal expansion and invasion. Biochim. Biophys. Acta Mol. Basis Dis..

[58] Zhang P., Zhong S., Wang G., Zhang S.Y., Chu C., Zeng S., Yan Y., Cheng X., Bao Y., Hocher B. (2018). N-Acetylcysteine Suppresses LPS-Induced Pathological Angiogenesis. Cell Physiol. Biochem..

[59] Panciera T., Citron A., Di Biagio D., Battilana G., Gandin A., Giulitti S., Forcato M., Bicciato S., Panzetta V., Fusco S. (2020). Reprogramming normal cells into tumour precursors requires ECM stiffness and oncogene-mediated changes of cell mechanical properties. Nat. Mater..

[60] Glienke J., Schmitt A.O., Pilarsky C., Hinzmann B., Weiss B., Rosenthal A., Thierauch K.H. (2000). Differential gene expression by endothelial cells in distinct angiogenic states. Eur. J. Biochem..

[61] Wang X., Freire Valls A., Schermann G., Shen Y., Moya I.M., Castro L., Urban S., Solecki G.M., Winkler F., Riedemann L. (2017). YAP/TAZ Orchestrate VEGF Signaling during Developmental Angiogenesis. Dev. Cell.

[62] Dupont S., Morsut L., Aragona M., Enzo E., Giulitti S., Cordenonsi M., Zanconato F., Le Digabel J., Forcato M., Bicciato S. (2011). Role of YAP/TAZ in mechanotransduction. Nature.

[63] Jiang K., Liu P., Xu H., Liang D., Fang K., Du S., Cheng W., Ye L., Liu T., Zhang X. (2020). SASH1 suppresses triple-negative breast cancer cell invasion through YAP-ARHGAP42-actin axis. Oncogene.

[64] Zhang Q., Jia Y., Pan P., Zhang X., Jia Y., Zhu P., Chen X., Jiao Y., Kang G., Zhang L. (2022). α5-nAChR associated with Ly6E modulates cell migration via TGF-β1/Smad signaling in non-small cell lung cancer. Carcinogenesis.

[65] Strusi G., Suelzu C.M., Weldon S., Giffin J., Munsterberg A.E., Bao Y. (2023). Combination of Phenethyl Isothiocyanate and Dasatinib Inhibits Hepatocellular Carcinoma Metastatic Potential through FAK/STAT3/Cadherin Signalling and Reduction of VEGF Secretion. Pharmaceutics.

[66] Han J.M., Jung H.J. (2022). Synergistic Anticancer Effect of a Combination of Berbamine and Arcyriaflavin A against Glioblastoma Stem-like Cells. Molecules.

[67] Huang Y.Q., Yuan J.D., Ding H.F., Song Y.S., Qian G., Wang J.L., Ji M., Zhang Y. (2018). Design, synthesis and pharmacological evaluation of a novel PEG-cRGD-conjugated irinotecan derivative as potential antitumor agent. Eur. J. Med. Chem..

[68] Richards C. (2020). Exploring New and Emerging Mechanisms to Target Difficult to Treat Cancers. Ph.D. Thesis.

[69] Sharma S., Alizadeh M., Pratt S., Stamatikos A., Abdelaziz K. (2025). Differential Expression of Key Immune Markers in the Intestinal Tract of Developing Chick Embryos. Vet. Sci..

[70] Martowicz A., Kern J., Gunsilius E., Untergasser G. (2015). Establishment of a Human Multiple Myeloma Xenograft Model in the Chicken to Study Tumor Growth, Invasion and Angiogenesis. J. Vis. Exp..

[71] Schulze J., Librizzi D., Bender L., Jedelska J., Yousefi B.H., Schaefer J., Preis E., Luster M., Mahnken A.H., Bakowsky U. (2023). How to Xenograft Cancer Cells on the Chorioallantoic Membrane of a Fertilized Hen’s Egg and Its Visualization by PET/CT and MRI. ACS Appl. Bio Mater..

[72] Herrmann A., Taylor A., Murray P., Poptani H., See V. (2018). Magnetic Resonance Imaging for Characterization of a Chick Embryo Model of Cancer Cell Metastases. Mol. Imaging.

[73] Sommerfeld S., Mundim A.V., Silva R.R., Queiroz J.S., Rios M.P., Notário F.O., Medeiros Ronchi A.A., Beletti M.E., Franco R.R., Espindola F.S. (2022). Physiological Changes in Chicken Embryos Inoculated with Drugs and Viruses Highlight the Need for More Standardization of this Animal Model. Animals.

[74] Ahmed T.A.E., Cordeiro C.M.M., Elebute O., Hincke M.T. (2022). Proteomic Analysis of Chicken Chorioallantoic Membrane (CAM) during Embryonic Development Provides Functional Insight. Biomed. Res. Int..

[75] McGrew M.J., Holmes T., Davey M.G. (2025). A scientific case for revisiting the embryonic chicken model in biomedical research. Dev. Biol..

[76] Handl V., Waldherr L., Arbring Sjöström T., Abrahamsson T., Seitanidou M., Erschen S., Gorischek A., Bernacka-Wojcik I., Saarela H., Tomin T. (2024). Continuous iontronic chemotherapy reduces brain tumor growth in embryonic avian in vivo models. J. Control. Release.

[77] Faihs L., Firouz B., Slezak P., Slezak C., Weissensteiner M., Ebner T., Ghaffari Tabrizi-Wizsy N., Schicho K., Dungel P. (2022). A Novel Artificial Intelligence-Based Approach for Quantitative Assessment of Angiogenesis in the Ex Ovo CAM Model. Cancers.

